# A rare pericytic tumor of the kidney: The first case in Korea

**DOI:** 10.1016/j.ijscr.2019.11.009

**Published:** 2019-11-09

**Authors:** Jae-Wook Chung, Jee Young Park, Heon Ha, Ki Bum Park, Yun-Sok Ha, Seock Hwan Choi, Jun Nyung Lee, Bum Soo Kim, Hyun Tae Kim, Tae-Hwan Kim, Eun Sang Yoo, Sung Kwang Chung, Ghil Suk Yoon, Tae Gyun Kwon

**Affiliations:** a Department of Urology, School of Medicine, Kyungpook National University, Daegu, Republic of Korea; b Department of Pathology, School of Medicine, Kyungpook National University, Daegu, Republic of Korea; c Department of Surgery, School of Medicine, Kyungpook National University, Daegu, Republic of Korea; d Joint Institute for Regenerative Medicine, Kyungpook National University, Daegu, Republic of Korea

**Keywords:** Pericytic, Tumor, Kidney

## Abstract

**Introduction:**

A pericytic tumor is a group of mesenchymal neoplasm found in superficial tissues and only rarely described in viscera. The family of pericytic tumors includes glomus tumors and variants, myopericytoma including myofibroma, and angioleiomyoma etc. The renal pericytic tumor is extremely rare, and only few comprehensive discussions about this entity have been done.

**Presentation of case:**

A 58-year-old man was transferred to our institute with suspicions of renal cell carcinoma. The kidney dynamic computed tomography scan showed a 3 cm sized solid mass in the upper pole of the right kidney. Laparoscopic radical nephrectomy was performed due to the deep-seated mass. Pathological result confirmed that the kidney mass was renal pericytic tumor.

**Discussion:**

Although general biological behavior of published renal pericytic tumors is likely to be benign, the clinicopathologic experiences are very limited. Therefore, we should evaluate the malignant potential of the entity according to the parameters proposed for soft tissue tumors, including tumor location, tumor size, growth pattern, cellularity, cytological atypia, and mitotic figures with atypical forms. The current case shows several worrisome features, including an extremely rare tumor location, partially infiltrative growth, and a mildly increased proliferating index, which resulted in it being classified as an uncertain malignant potential.

**Conclusion:**

We described the first case of renal pericytic tumor, addressing uncertain malignant potential, in a Korean male, which would be a distinct mesenchymal neoplasm differentiating from other groups of perivascular tumor families based on histological and immunohistochemical features.

## Highlights

• The family of pericytic tumors includes glomus tumors and variants, myopericytoma including myofibroma, and angioleiomyoma.
• The renal pericytic tumor is extremely rare, and only few comprehensive discussions about this entity have been done.
• We report the first documented case of renal pericytic tumor in a 58-year-old Korean male.

## 1 Introduction

Pericytic (perivascular) tumors are distinct mesenchymal neoplasms that rarely involve the kidney. Instead, pericytic tumors are morphologically related to the differentiation of perivascular myoid cells which invest blood vessels and function physiologically in vascular modification and thermoregulation. These include myopericytoma, myofibroma, angioleiomyoma, glomus tumors and variants [1].

The latest WHO classification of kidney tumors [2] does not address the pericytic tumor since the tumor has been exceptionally rare and has been limited in experiences for the risk prediction of clinical behaviors. Herein, we describe the first case of pericytic tumor of the kidney, considering its uncertain malignant potential, in a Korean male, and we offer a brief comment. This work has been reported in line with the SCARE criteria [3].

## 2 Case report

### 2.1 Case presentation

**Fig. 1:**
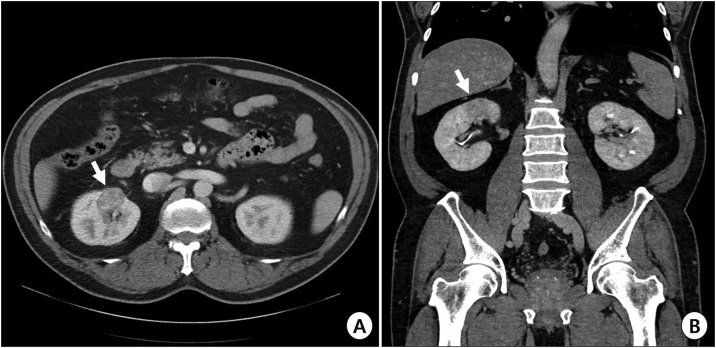
Preoperative kidney dynamic computed tomography scan. a) Axial view. b) Coronal view. Arrows indicate the mass lesion located in the right kidney upper pole mimicking renal cell carcinoma.

A 58-year-old man underwent abdominal ultrasonography for a health screening at a local clinic. The patient was transferred to our institute with suspicions of renal cell carcinoma. The kidney dynamic computed tomography scan showed a 3 cm sized solid mass in the upper pole of the right kidney (Fig. 1A–B). He had no previous medical history barring ureteric stones. The level of serum creatinine was within normal range. Laparoscopic radical nephrectomy was performed due to the deep-seated mass.

### 2.2 Pathological findings

**Fig. 2:**
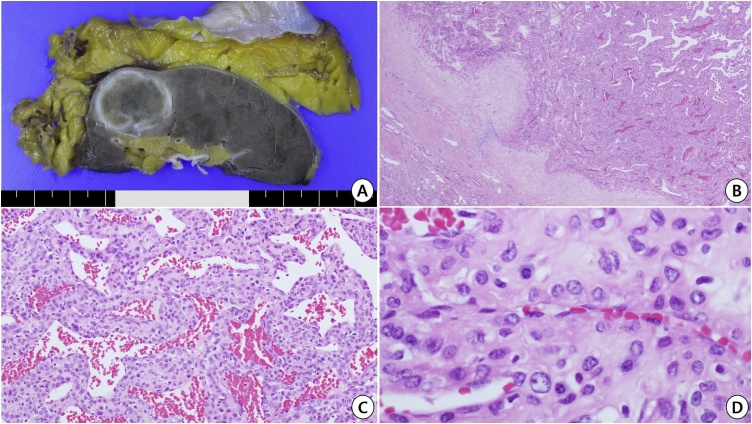
Gross and histopathologic features in this case. a) Gross examination. b) Partial capsular invasion of the pericytic tumor (H&E stain, ×20). c) Perivascular arrangement of tumor cells (H&E, ×100). d) Nuclear feature of tumor cells (H&E, ×400).

On gross examination, there was a well-defined, pale-tan-colored, round and solid mass in the upper portion of the right kidney measuring 3.2 × 2.2 cm (Fig. 2A). There was neither hemorrhage nor necrosis on the cut surface. The tumor was confined to the kidney; there was no invasion into the renal capsule.

**Fig. 3:**
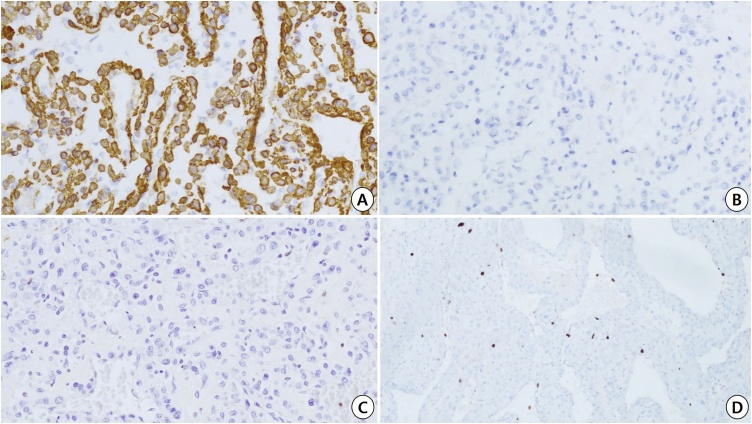
Immunohistochemical findings in this case. a) Immunopositive feature for smooth muscle actin (×200). b) Immunonegativity for cytokeratin (×200). c) Immunonegativity for HMB-45 (×200). d) Ki-67 labeling index of tumor cells (×100).

Microscopically, the tumor showed an irregularly circumferential fibrotic capsule with multifocal capsular infiltrations (Fig. 2B). Tumor cells were arranged as a mixture of compactly nesting, perivascular, and hemangiopericytoma-like patterns, often showing concentric manners around muscular vessels (Fig. 2C). Tumor cells had bland-looking, oval-to-short-spindle nuclei with evenly fine chromatin and occasional distinct nucleoli, and abundant eosinophilic cytoplasm (Fig. 2D). Mitotic figures were observed at two per 50 high-power field without atypical forms. No necrosis was identified. The intervening stroma showed variable degenerative changes including stromal edema and hyalinization. Immunohistochemically, tumor cells were positive for smooth muscle actin (Fig. 3A) and vimentin; while, they were negative for cytokeratin (Fig. 3B), desmin, CD10, CD31, CD34, S-100 protein, HMB-45 (Fig. 3C), and Melan-A. Ki-67 labeling index was 7.8 % by morphometric analysis with GenASIs Hipath system (Applied Spectral Imaging, Carlsbad, California, USA) (Fig. 3D).

## 3 Discussion

The pericytic (perivascular) tumors are mesenchymal neoplasms rarely encountered in visceral sites, but are considered to be same entities to their counterparts in skin and soft tissue because of morphological similarities [1]. The World Health Organization (WHO) classification of tumors of soft tissue defines a group of pericytic (perivascular) tumors including glomus tumors and variants, myopericytoma, myofibroma, and angioleiomyoma [4]; however, the latest WHO classification of kidney tumors does not list pericytic tumors since those tumors were exceptionally rare in the kidney [2].

The current case considering the epithelioid morphology with characteristic vascularity should be differentiated from perivascular epithelioid cell tumor (PEComa), glomus tumor, juxtaglomerular cell tumor (JGCT), epithelioid solitary fibrous tumor (SFT), carcinoid tumor, and clear cell renal cell carcinoma (CCRCC). PEComas are often composed of triphasic components of angiomyolipoma, and they are immunopositive for melanocytic makers, such as HMB-45 and Melan-A. Glomus tumors consist of more rounded small tumor cells, but relatively lack the concentric orientation of tumor cells around vessels [5]. JGCTs usually occur in adolescents and young adults, and present with hypertension correlated to plasma renin activity [1]. The tumors demonstrate nested small round cells in vascular backgrounds with pathognomic rhomboid-shaped renin crystals on ultrastructural examination, and immunoreactivities for CD34 and CD117 [1]. SFTs are considered to be fibroblastic/myofibroblastic in origin, and are recently characterized of STAT6 immunopositivity resulting in NAB2/STAT6 in tumorigenesis [6,7]. Carcinoid tumors show nested epithelioid histology with immunopositivities for neuroendocrine markers. CCRCC with low histologic grade can be separated by morphologic features and immunohistochemical stains, e.g. cytokeratin, EMA, CD10, and carbonic anhydrase IX etc [1,8].

Although general biological behavior of published renal pericytic tumors is likely to be benign, the clinicopathologic experiences are very limited. Therefore, we should evaluate the malignant potential of the entity according to the parameters proposed for soft tissue tumors, including tumor location, tumor size, growth pattern, cellularity, cytological atypia, and mitotic figures with atypical forms. The current case shows several worrisome features, including an extremely rare tumor location, partially infiltrative growth, and a mildly increased proliferating index, which resulted in it being classified as an uncertain malignant potential.

In summary, we described the first case of renal pericytic tumor, addressing uncertain malignant potential, in a Korean male, which would be a distinct mesenchymal neoplasm differentiating from other groups of perivascular tumor families based on histological and immunohistochemical features.

## Sources of funding

No sources to be stated.

## Ethical approval

This study was approved by the Ethics Committee of the Kyungpook National University School of Medicine (IRB Number KNUH 2019-08-004).

## Consent

Written informed consent was obtained from the patient for publication of this case report.

## Author’s contribution

Jae-Wook Chung: Surgeon of the patient’s procedure described in the case report, concept and design of study.

Jee Young Park, Heon Ha, Ki Bum Park, Yun-Sok Ha, Seock Hwan Choi, Jun Nyung Lee, Bum Soo Kim, Hyun Tae Kim, Tae-Hwan Kim, Eun Sang Yoo, Sung Kwang Chung: Writing the manuscript.

Ghil Suk Yoon, Tae Gyun Kwon: Approving the final version of the manuscript.

## Registration of research studies

researchregistry5193.

## Guarantor

Jae-Wook Chung.

## Provenance and peer review

Not commissioned, externally peer-reviewed.

## Declaration of Competing Interest

There is no conflict to be declared.
